# Bevacizumab with preoperative chemotherapy versus preoperative chemotherapy alone for colorectal cancer liver metastases: a retrospective cohort study

**DOI:** 10.1097/MD.0000000000004767

**Published:** 2016-09-02

**Authors:** Zhen-Hai Lu, Jian-Hong Peng, Fu-Long Wang, Yun-Fei Yuan, Wu Jiang, Yu-Hong Li, Xiao-Jun Wu, Gong Chen, Pei-Rong Ding, Li-Ren Li, Ling-Heng Kong, Jun-Zhong Lin, Rong-Xin Zhang, De-Sen Wan, Zhi-Zhong Pan

**Affiliations:** a Department of Colorectal Surgery; b Department of Hepatobiliary Surgery; c Department of Medical Oncology, Sun Yat-sen University Cancer Center; State Key Laboratory of Oncology in South China; Collaborative Innovation Center of Cancer Medicine, Guangzhou, PR China.

**Keywords:** bevacizumab, colorectal cancer, liver metastases, preoperative chemotherapy

## Abstract

This study aimed to assess the efficacy and safety of bevacizumab plus preoperative chemotherapy as first-line treatment for liver-only metastatic colorectal cancer in Chinese patients compared with those of preoperative chemotherapy alone.

Patients with histologically confirmed liver-only metastatic colorectal cancer were sequentially reviewed, and received either preoperative chemotherapy plus bevacizumab (bevacizumab group, n = 32) or preoperative chemotherapy alone (chemotherapy group, n = 57). Progression-free survival, response rate, liver resection rate, conversion rate, and safety were analyzed.

With median follow-up of 28.7 months, progression-free survival was 10.9 months (95% confidence interval: 8.7–13.1 months) in bevacizumab group and 9.9 months (95% confidence interval: 6.8–13.1 months) in chemotherapy group (*P* = 0.472). Response rates were 59.4% in bevacizumab group and 38.6% in chemotherapy group (*P* = 0.059). Overall liver resection (R0, R1, and R2) rate was 68.8% in bevacizumab group and 54.4% in chemotherapy group (*P* = 0.185). Conversion rate was 51.9% in bevacizumab group and 40.4% in chemotherapy group (*P* = 0.341). No postoperative complication was observed in all patients.

Bevacizumab plus preoperative chemotherapy as first-line treatment for liver-only metastatic colorectal cancer tends to achieve better clinical benefit with controllable safety in Chinese patients.

## 1 Introduction

Colorectal cancer (CRC) is one of the most common cancers, with a total of 143,460 new cancer cases and 51,690 deaths from cancer being projected to occur in the United States in 2012.^[1]^ The development of metastatic disease from CRC is the major leading cause of death. Liver is the most common site of CRC metastasis. At the time of diagnosis, 25% patients with CRC have liver metastases and additional 25% to 45% patients develop liver metastases with disease progression. These patients often have poor prognosis, despite advances in chemotherapy.^[2]^ However, a proportion of patients considered ineligible for liver resection would achieve sufficient tumor response after a period of conversion chemotherapy to allow liver resection and this group of patients subsequently can achieve a respectable survival benefit.^[3]^

Bevacizumab is a humanized monoclonal antibody targeting vascular endothelial growth factor and thus inhibiting its interaction with vascular endothelial growth factor receptor. Two randomized phase III trials reported that the addition of bevacizumab to chemotherapy was associated with improved median progression-free survival (mPFS) in patients with metastatic CRC (mCRC).^[4,5]^ Furthermore, several studies have found that the addition of bevacizumab to chemotherapy could improve response rate and the rate of liver resection in patients with mCRC.^[2,6]^ However, the clinical outcome of the addition of bevacizumab to commonly used chemotherapy in Chinese patients with liver-only mCRC remains unclear.

This retrospective study aims to evaluate the efficacy and safety of bevacizumab plus preoperative chemotherapy compared with those of preoperative chemotherapy alone as first-line treatment for liver-only metastases CRC in Chinese patients.

## 2 Methods

### 2.1 Patients

We reviewed the electronic medical records of patients who sequentially received treatment in Sun Yat-sen University Cancer Center, Guangzhou, China, from May 15, 2009, to June 27, 2013. Patients met the following criteria: histologically proven colorectal adenocarcinomas, abdominal/pelvic computerized tomography (CT) or magnetic resonance imaging (MRI) diagnosed initially resectable or initially unresectable hepatic metastases, age between 18 and 75 years, Eastern Cooperative Oncology Group performance status ≤2,^[7]^ no evidence of other metastatic lesions except for the liver based on abdominal/pelvic/chest CT, and adequate hematological, liver, and renal functions. Patients with locally advanced rectal cancer underwent concurrent chemoradiotherapy and then received primary tumor resection before metastases treatment. Patients were excluded if they had the following conditions: history of other active malignancy (except for basal cell carcinoma of the skin) during previous 5 years, uncontrolled severe cardiovascular and respiratory system diseases, chemotherapy, treatment with aspirin or nonsteroidal anti-inflammatory medications, and pregnancy. The study was done in accordance with the ethical standards of the World Medical Association Declaration of Helsinki. Waiver of informed consent was requested, and the study approval was obtained from Ethics Committee of Sun Yat-sen University Cancer Center.

### 2.2 Treatment

The patients received preoperative chemotherapy with or without bevacizumab according to the decision of multidisciplinary team including staffs from Department of Colorectal Surgery, Hepatobiliary Surgery, Medical Oncology, Medical Imaging and Invasive Technology. Strategy of preoperative chemotherapy was decided based on clinical evaluation by the oncologist. There were 2 preoperative chemotherapy regimens: FOLFOX (oxaliplatin 85 mg/m^2^ and leucovorin 400 mg/m^2^ were administered intravenously over 2 hours on the first day, 5-FU (5-fluorouracil) 400 mg/m^2^ injected intravenously on the first day and then 1200 mg/m^2^ administered intravenously for 2 days for 2-week cycle) and FOLFIRI (irinotecan 180 mg/m^2^ and leucovorin 400 mg/m^2^ were administered intravenously over 2 hours on the first day, 5-FU 400 mg/m^2^ was injected intravenously on the first day and then 1200 mg/m^2^ administered intravenously for 2 days for 2-week cycle). Bevacizumab 5 mg/kg was administered intravenously on the first day every 2 weeks combined with FOLFOX or FOLFIRI regimen. Operability of liver metastasis was assessed by CT and MRI scans every 6 weeks. Patients deemed to be resectable would undergo surgery with at least 6-week interval from the last dose of bevacizumab and 4 weeks from the last dose of chemotherapy. Patients who remained unresectable were suggested to receive the following treatment strategies: radiofrequency ablation, microwave treatment, transarterial chemoembolization, and continuation of chemotherapy. The same chemotherapy regimen was applied after 3 weeks following operation. Chemotherapy continued until disease progression and halted if severe adverse events occurred. For follow-up, patients were observed through telephone interview or subsequent visit every 3 months until death or loss to follow-up.

### 2.3 Study assessment

All patients underwent an assessment of tumor status at baseline and every 6 weeks after chemotherapy by abdominal/pelvic/chest CT and MRI. Tumor response or progression was determined according to the Response Evaluation Criteria in Solid Tumors.^[8]^ Patients without tumor assessments were categorized as nonresponders. Conversion was defined as initially unresectable liver metastasis converted into R0 resectable lesion after preoperative chemotherapy. Safety was assessed by reports of adverse events and clinical laboratory test results. Adverse events and abnormal laboratory results were graded utilizing the National Cancer Institute Common Toxicity Criteria, version 3.0.^[9]^ Information on postoperative complications was obtained from electronic medical records of patients after surgery.

### 2.4 Statistical analysis

Progression-free survival (PFS), liver metastases response rate (complete and partial), liver resection rate (R0, R1, and R2), conversion rate, and safety were analyzed. PFS was defined as the time from the beginning of preoperative chemotherapy to the disease progression or death at the cutoff time. For patients alive without disease progression at the time of analysis, PFS was censored on the date of the last follow-up. Statistical analyses were performed with SPSS (version 17.0; SPSS Inc, Chicago, IL). Continuous variables were summarized by median (range) and categorical variables were presented by percentage. Continuous variable was analyzed using *t* test and categorical variable was analyzed using χ^2^ test or Fisher test, as appropriate. Kaplan–Meier analysis was applied to evaluate mPFS and mPFS between the 2 groups were compared with the log-rank test. *P* < 0.05 was considered significant.

## 3 Results

### 3.1 Patients’ characteristics

Eighty-nine eligible patients were reviewed in our study. Thirty-two patients received preoperative chemotherapy plus bevacizumab (bevacizumab group), and 57 patients were treated with preoperative chemotherapy alone (chemotherapy group). Patients and disease characteristics at baseline were generally well balanced between the 2 groups (Table 1).

**Table 1:**
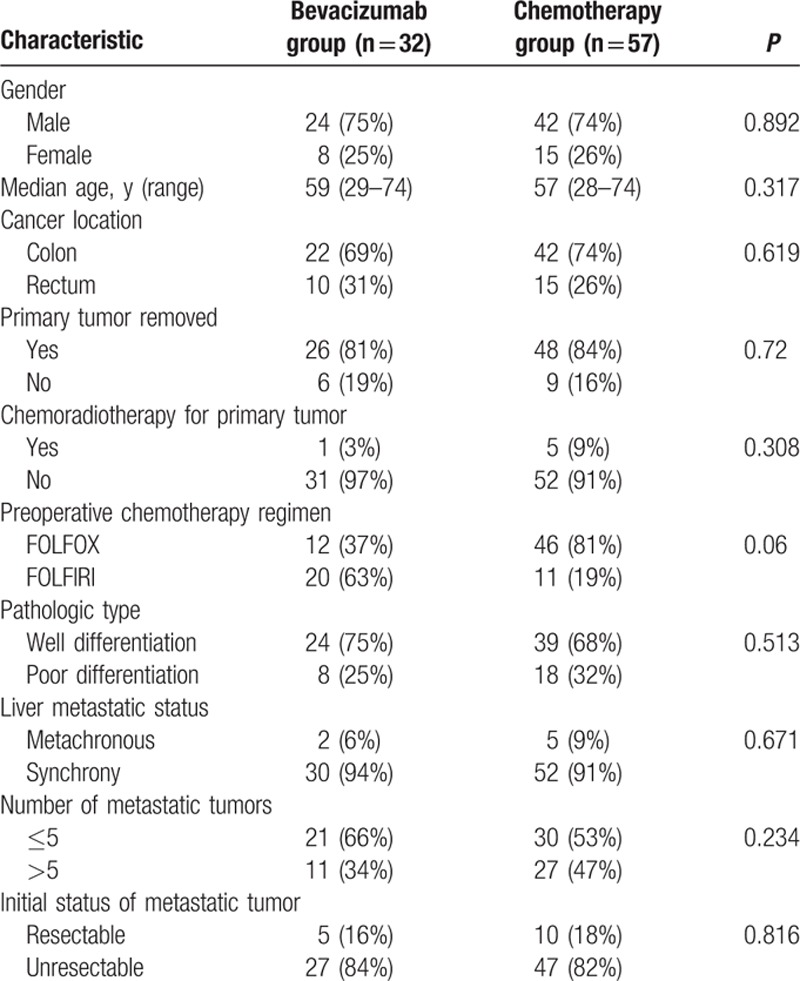
Baseline characteristics of patients.

### 3.2 Chemotherapy treatment

From the start of chemotherapy, the median duration of chemotherapy was 12 weeks (range 6–20 weeks) in bevacizumab group and 10 weeks (range 4–24 weeks) in chemotherapy group. Bevacizumab was administered for a median of 8 weeks (range 4–18 weeks).

### 3.3 Efficacy

**Figure 1:**
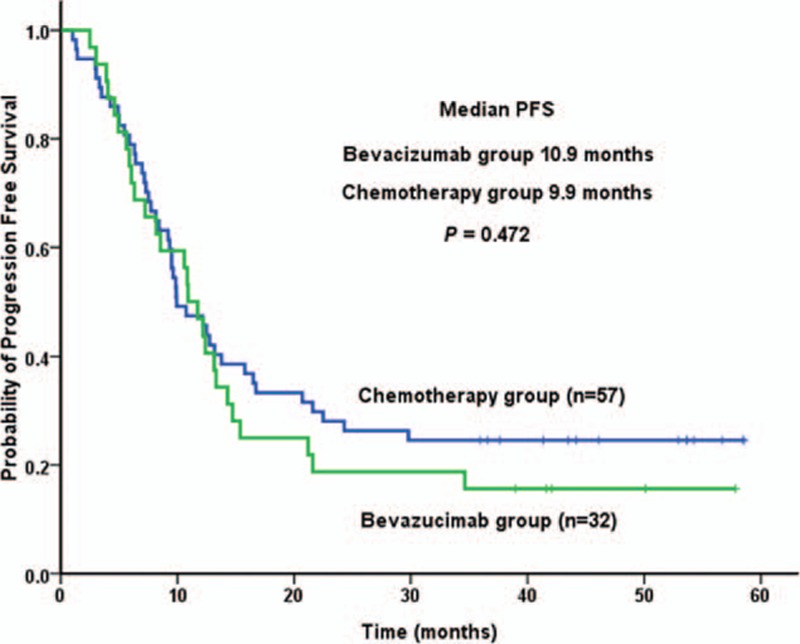
Kaplan–Meier curve for PFS of patients treated with bevacizumab plus chemotherapy or chemotherapy alone. PFS = progression-free survival.

At the clinical cutoff date of June 6, 2016, all patients were recruited for follow-up. With median follow-up of 28.7 months (range 5–70 months), mPFS was 10.9 months (95% confidence interval: 8.7–13.1 months) in bevacizumab group and 9.9 months (95% confidence interval: 6.8–13.1 months) in chemotherapy group (*P* = 0.472). Longer mPFS was observed in bevacizumab group, as shown in Table 2 and Fig. 1. No significant difference in mPFS was observed between the 2 groups by comparison according to several clinical parameters (Table 3).

**Table 2:**
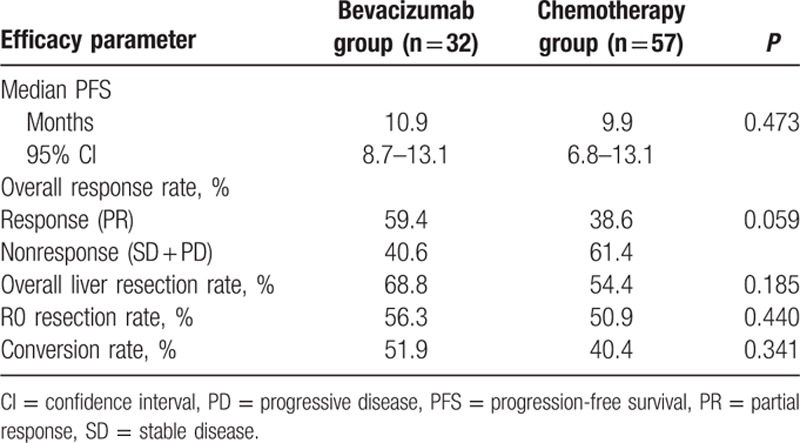
Summary of efficacy in both groups.

**Table 3:**
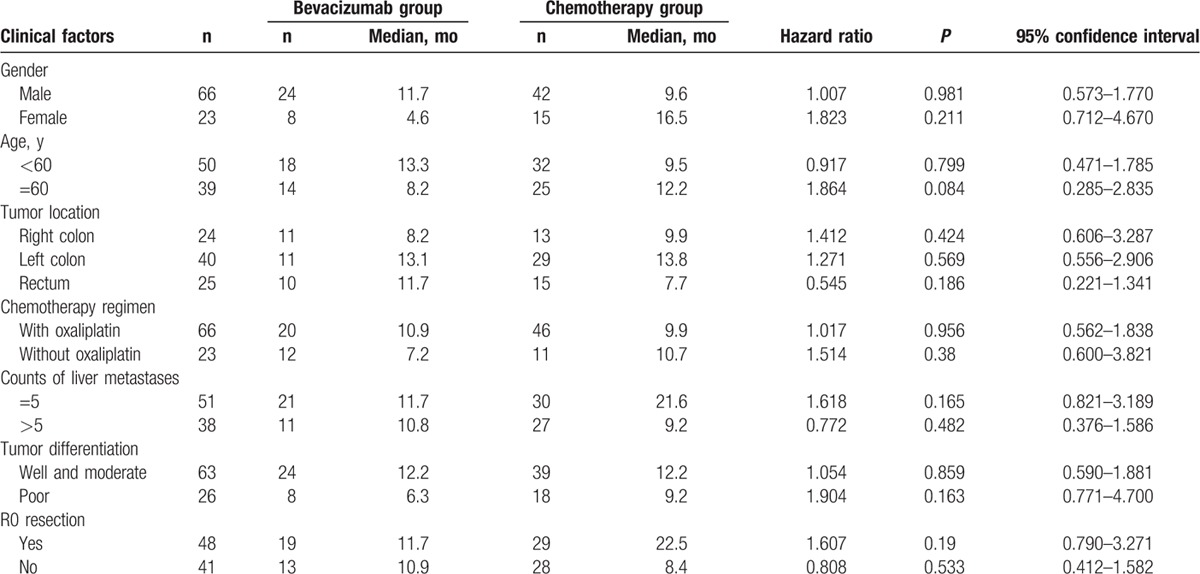
Univariate analysis of clinical factors for progression-free survival.

Two patients without tumor assessment were categorized as nonresponders. No patient achieved complete response during treatment in the 2 groups. Patients treated with bevacizumab plus preoperative chemotherapy had higher partial response rate compared to chemotherapy group (59.4% vs 38.6%, *P* = 0.059; Table 2). In bevacizumab group, 13 (40.6%) patients had stable disease; in chemotherapy group, 29 (50.9%) patients had stable disease and 6 (10.5%) had progressive disease.

For treatment, in bevacizumab group, R0 resection was performed in 18 (56.3%) patients, R1 resection in 1 (3.1%) patient, R2 resection in 1 (3.1%) patient, radiofrequency ablation in 2 (6.3%) patients, microwave treatment in 3 (9.4%) patients, and the remaining 7 (21.9%) patients received no treatment; in chemotherapy group, R0 resection was performed in 29 (50.9%) patients, R1 resection in 2 (3.5%) patients, transarterial chemoembolization in 4 (7.0%) patients, and the remaining 22 (36.6%) patients received no treatment.

There was no significant difference in the overall liver resection (R0, R1, and R2 resection) rate (68.8% vs 54.4%, *P* = 0.185) and R0 resection rate (56.3% vs 50.9%, *P* = 0.440) between the 2 groups (Table 2). In bevacizumab group, 14 of 27 patients with initially unresectable metastasis achieved R0 resection; the rate was higher than in chemotherapy group (51.9% vs 40.4%, *P* = 0.341; Table 2). Furthermore, FOLFOX regimen plus bevacizumab subgroup showed a higher conversion rate compared with FOLFIRI regimen plus bevacizumab subgroup (59% vs 40%, *P* = 0.44; Table 3).

**Figure 2:**
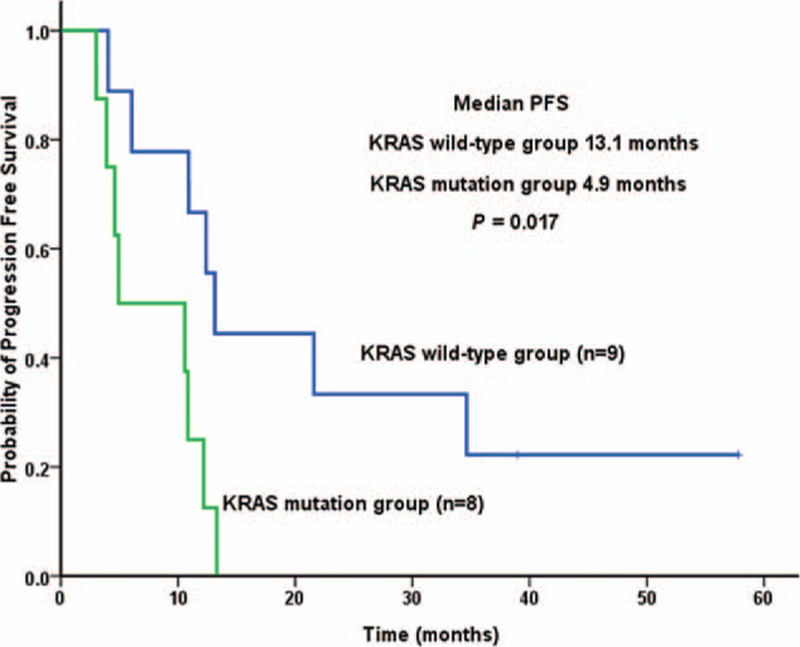
Kaplan–Meier curve for PFS of patients with *KRAS* wild-type or *KRAS* mutation in bevacizumab group. PFS = progression-free survival.

The clinical results of bevacizumab cohort were compared by subgroups (Table 4). *KRAS* gene status was detected in 17 cases. Patients with *KRAS* mutation had worse PFS compared to those with wild-type *KRAS* (4.9 vs 13.1 months, *P* = 0.017; Fig. 2). Chemotherapy regimen and primary tumor location were not associated with clinical outcome in bevacizumab group.

**Table 4:**
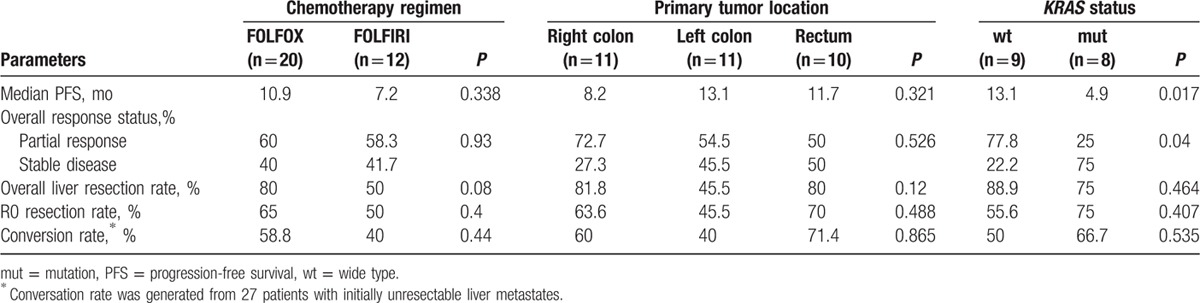
Comparison of clinical outcome of the subgroups in bevacizumab cohort.

### 3.4 Safety

The incidence of treatment-related adverse events was similar between the 2 groups (Table 5). Overall adverse events occurred in 4 (12.5%) of 32 patients in bevacizumab group and 5 (8.8%) of 57 in chemotherapy group. For grade 3 or worse adverse events, 1 case (3.1%) of leukopenia occurred in bevacizumab group; 2 (3.5%) patients experienced thrombocytopenia and 1 experienced leukopenia (1.8%) in preoperative chemotherapy group. Only 1 patient (3.1%) experienced grade 3 bleeding gums after treatment with bevacizumab. No postoperative complications were observed in chemotherapy or bevacizumab group. No patients died due to the treatment.

**Table 5:**
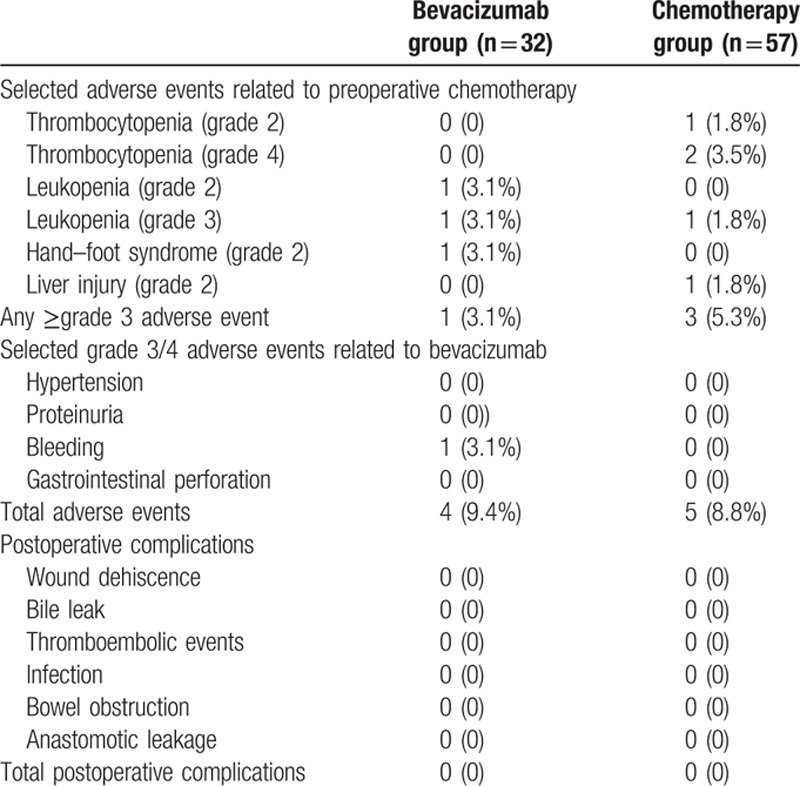
Selected adverse events and postoperative complications in both cohorts.

## 4 Discussion

The present study showed that the addition of bevacizumab to preoperative chemotherapy may potentially achieve higher clinical efficacy (PFS, response rate, and conversion rate) with manageable toxicities compared to preoperative chemotherapy in Chinese patients with liver-only mCRC.

Previous studies have shown that the addition of bevacizumab to preoperative chemotherapy significantly improved PFS but not overall survival compared with preoperative chemotherapy alone in mCRC.^[5,10,11]^ However, few studies reported the result of long-term outcome related to bevacizumab for patients with liver-only mCRC. The present study showed that the mPFS of patients who received bevacizumab plus chemotherapy tended to be improved by one month compared to that of patients who received chemotherapy alone (10.9 vs 9.9 months), although there was no statistically significant difference between the 2 groups.

Several studies have evaluated the response of CRC liver metastases to preoperative chemotherapy combined with bevacizumab. A phase II study reported that response rate was 66.7% after 6-cycle neoadjuvant FOLFIRI + bevacizumab treatment in patients with resectable liver metastases from CRC.^[12]^ Compared to chemotherapy alone, the addition of bevacizumab improved liver metastases response rate.^[3,13,14]^ In the present study, we evaluated the response of colorectal metastases by using imaging technology and confirmed that the addition of bevacizumab can lead to higher response rate compared with preoperative chemotherapy alone (59.4% vs 38.6%). For patients with initially unresectable liver metastases, a strong correlation between response rate and resection rate in neoadjuvant treatment of mCRC was demonstrated.^[15]^ In our study, the addition of bevacizumab tended to convert initially unresectable liver metastasis into R0 resection, although the results failed to reach statistical significance (51.9% vs 40.4%, *P* = 0.341). A multicenter study demonstrated that neoadjuvant chemotherapy plus bevacizumab resulted in a high response rate for patients with mCRC and converted 40% patients to resectability.^[6]^ However, another study reported that the addition of bevacizumab to preoperative chemotherapy could not improve R0 metastasectomy.^[16]^ We consider that the initial selection of patients can be an important factor leading to the controversial results of these studies. Patients with potentially resectable metastases may easily achieve R0 metastasectomy compared to those with unresectable metastases. Furthermore, different chemotherapy regimen may lead to different liver resection rate. Our study showed that bevacizumab combined with oxaliplatin-based chemotherapy led to significantly higher liver resection rate than bevacizumab with non–oxaliplatin-based chemotherapy (80% vs 50%, *P* = 0.08). Several studies have demonstrated that bevacizumab plus oxaliplatin-based chemotherapy could achieve a favorable liver resection rate from 47.8% to 92.8%.^[2,6,17]^

Addition of bevacizumab raises safety concerns regarding increased risk of serious adverse events.^[18,19]^ Bevacizumab can increase the risk of hypertension, proteinuria, bleeding, and thromboembolic events.^[5]^ However, only 1 patient developed grade 3 bleeding gums associated with bevacizumab in our study. The incidences of adverse events reported in this study were lower than those reported in previous randomized trials.^[20,21]^ We consider that the small volume sample of this study may attribute to less adverse events observed in patients treated with bevacizumab. In addition, in our study bevacizumab was administered for a median of 8 weeks (range 4–18 weeks), shorter than the time in previous trials.^[5]^ Bevacizumab is known to cause various postoperative complications such as thrombosis, late anastomotic complications, gastrointestinal perforation, and delayed wound healing.^[22,23]^ However, no postoperative complication was observed in our study. A retrospective study on mCRC undergoing liver resection reported that the rate of postoperative complications showed no significant difference between chemotherapy group and bevacizumab group.^[24]^ These data suggest that bevacizumab can be safely administered in patients with liver-only mCRC without increasing the rate of adverse events or postoperative complications.

There are several limitations in the current study. First, the small sample of this study and inadequate treatment cycles of bevacizumab (median 8 weeks) may contribute to no statistical significance of the results. Second, the time of follow-up is too short to evaluate overall survival; thus, we failed to compare overall survival between the 2 groups. Third, patients were treated with various preoperative chemotherapy regimens, which can lead to systematic bias.

In conclusion, our study suggests that the addition of bevacizumab to preoperative chemotherapy may potentially improve PFS and increase R0 resection rate of initially unresectable liver-only metastases in patients with CRC with controllable toxicities. In addition, bevacizumab with oxaliplatin-based regimen may achieve a favorable clinical outcome.
